# Supplementary material for: Core-Shell Structured SiO2@NiFe LDH Composite for Broadband Electromagnetic Wave Absorption
Source: Int J Mol Sci. 2022 Dec 28;24(1):504. doi: 10.3390/ijms24010504 (PMC9820398; doi:10.3390/ijms24010504)
Supplement: Supplementary file 1 [file ijms-24-00504-s001.zip › ijms-2096978-supplementary.pdf]

Supplementary Material

Core-Shell Structured SiO_2@NiFe LDH Composite for Broadband Electromagnetic Wave Absorption

Zhilan Du [1,†], Dashuang Wang [1,†], Xinfang Zhang [1], Zhiyu Yi [1], Jihai Tang [2], Pingan Yang [3], Rui Cai [3], Shuang Yi [1], Jinsong Rao [1] and Yuxin Zhang [1,*]

[1] College of Material Science and Engineering, Chongqing University, Chongqing 400044, China
[2] No. 59 Research Institute of China Ordnance Industries, Chongqing 400039, China
[3] School of Automation, Chongqing University of Posts and Telecommunications, Chongqing 400065, China
* Correspondence: zhangyuxin@cqu.edu.cn
† These authors contributed equally to this work.

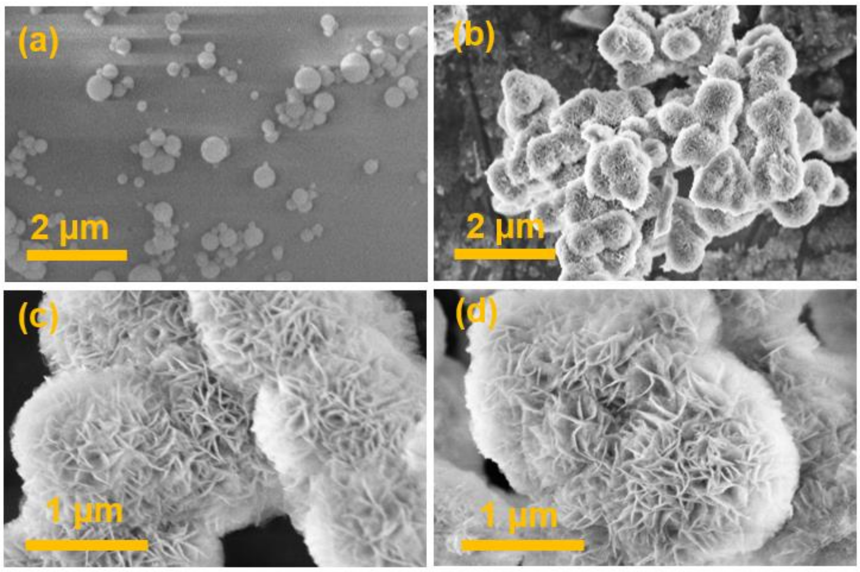

Figure S1. (**a**) SEM image of SiO_2; (**b-d**) the enlarged images of SiO_2@NiFe-LDH-3.

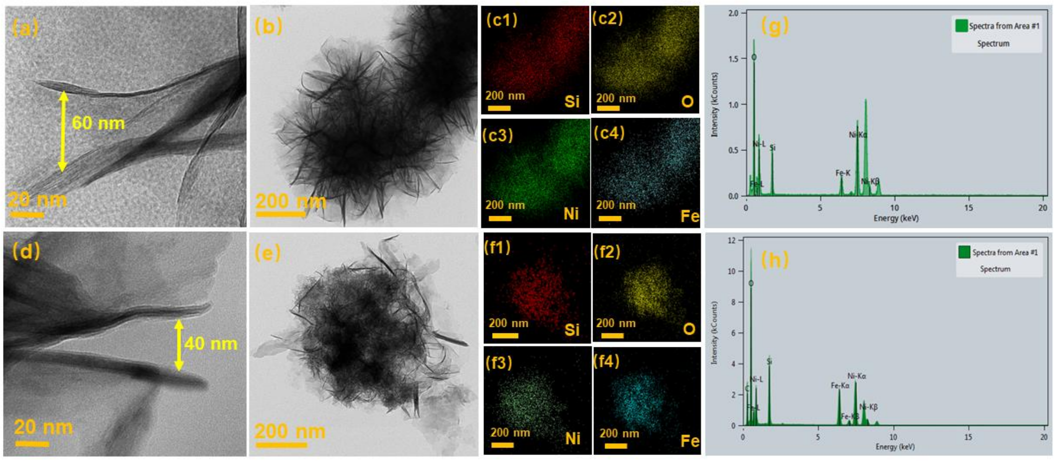

Figure S2. TEM image of (a) the enlarged detail of SiO_2@NiFe LDH-1; (b) SiO_2@NiFe LDH-1; (c1-c4) the EDS Mapping of SiO_2@NiFe LDH-1; (d) the enlarged detail of SiO_2@NiFe LDH-2; (e) SiO_2@NiFe LDH-2; (f1-f4) the EDS Mapping of SiO_2@NiFe LDH-2; (g) EDS spectrum of SiO_2@NiFe LDH-1; (h) EDS spectrum of SiO_2@NiFe LDH-2.

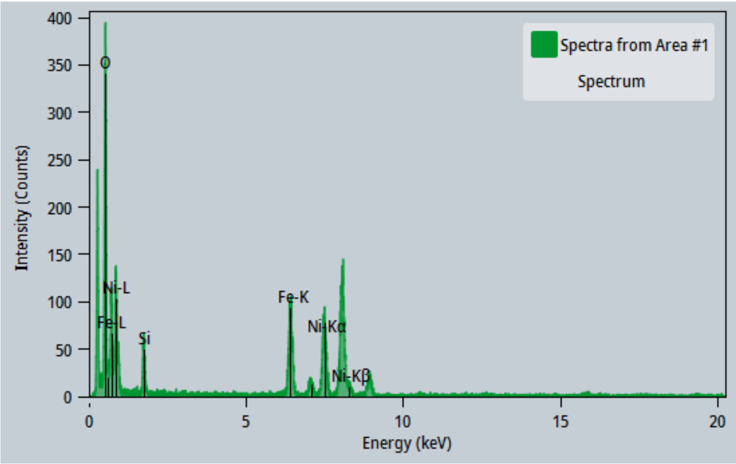

Figure S3. EDS spectrum of SiO_2@NiFe LDH-3.

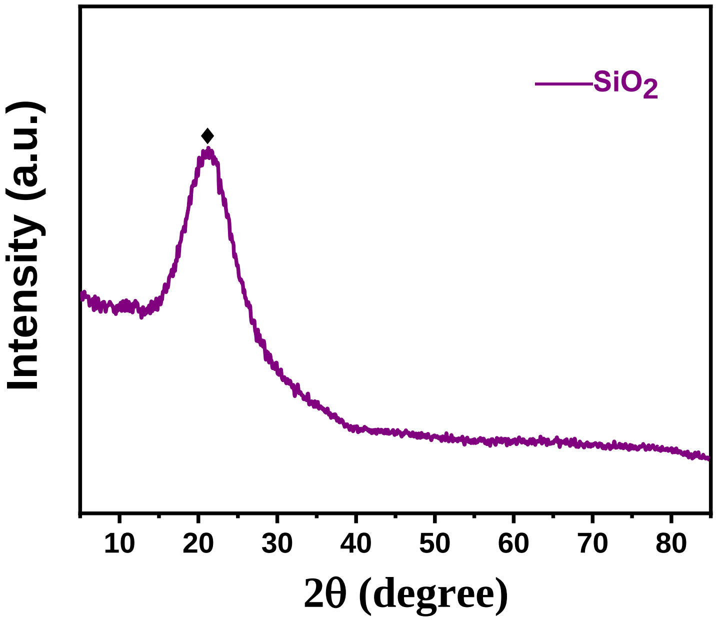

Figure S4. The XRD pattern of SiO_2.

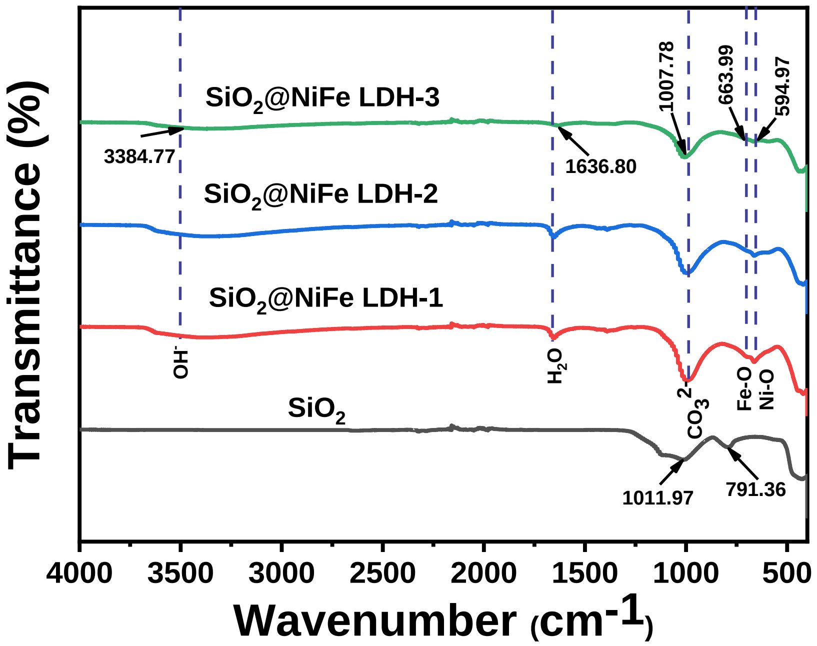

Figure S5. FT-IR spectra of SiO_2 and SiO_2@NiFe LDH-1, 2, 3.

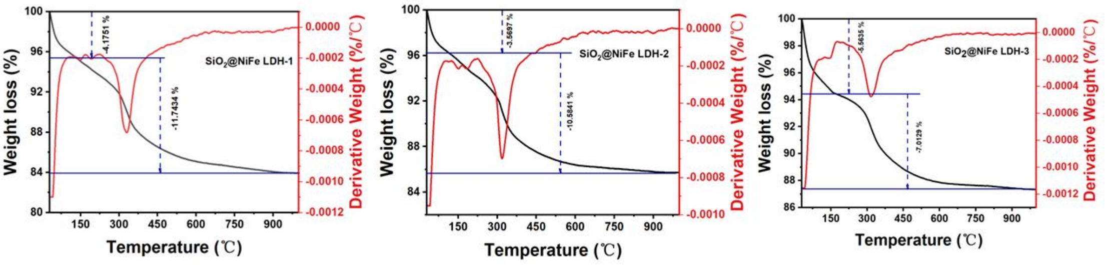

(**a**) (**b**) (**c**)

Figure S6. The TGA of (**a**) SiO_2@NiFe LDH-1, (**b**) SiO_2@NiFe LDH-2, (**c**) SiO_2@NiFe LDH-3.

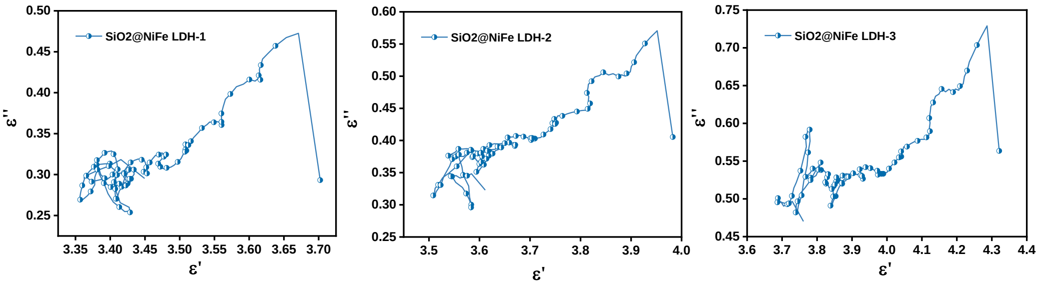

(**a**) (**b**) (**c**)

Figure S7. Cole-Cole semicircles of (**a**) SiO_2@NiFe LDH-1, (**b**) SiO_2@NiFe LDH-2 and (**c**) SiO_2@ NiFe LDH-3.
